# Diversity of the DNA Replication System in the *Archaea* Domain

**DOI:** 10.1155/2014/675946

**Published:** 2014-03-26

**Authors:** Felipe Sarmiento, Feng Long, Isaac Cann, William B. Whitman

**Affiliations:** ^1^Department of Microbiology, University of Georgia, 541 Biological Science Building, Athens, GA 30602-2605, USA; ^2^3408 Institute for Genomic Biology, University of Illinois, 1206 W Gregory Drive, Urbana, IL 61801, USA

## Abstract

The precise and timely duplication of the genome is essential for cellular life. It is achieved by DNA replication, a complex process that is conserved among the three domains of life. Even though the cellular structure of archaea closely resembles that of bacteria, the information processing machinery of archaea is evolutionarily more closely related to the eukaryotic system, especially for the proteins involved in the DNA replication process. While the general DNA replication mechanism is conserved among the different domains of life, modifications in functionality and in some of the specialized replication proteins are observed. Indeed, *Archaea* possess specific features unique to this domain. Moreover, even though the general pattern of the replicative system is the same in all archaea, a great deal of variation exists between specific groups.

## 1. Introduction

*Archaea* are a diverse group of prokaryotes which are united by a number of unique features. Many of these microorganisms normally thrive under extreme conditions of temperature, salinity, pH, or pressure. However, some also coexist in moderate environments with* Bacteria* and eukaryotes [1, 2]. Likewise, a wide range of lifestyles, including anaerobic and aerobic respiration, fermentation, photo- and chemoautotrophy, and heterotrophy, are common among this group. In spite of this diversity, members of the* Archaea* domain possess specific physiological characteristics that unite them, such as the composition and structure of their membrane lipids [3].

*Archaea*, for the most part, resemble* Bacteria* in size and shape, but they possess important differences at genetic, molecular, and metabolic levels. Remarkable differences are present in the machinery and functionality of the information processing systems. For example, the histones in some* Archaea* [4], the similarity of proteins involved in transcription and translation, and the structure of the ribosome are more closely related to those of eukaryotes than* Bacteria* [5]. The most striking similarities between* Archaea* and eukaryotes are observed in DNA replication, one of the most conserved processes in living organisms, where the components of the replicative apparatus and the overall process closely resemble a simplified version of the eukaryotic DNA replication system [6, 7]. However, a few characteristics of the archaeal information processing system are shared only with* Bacteria*, such as the circular topology of the chromosome, the small size of the genome, and the presence of polycistronic transcription units and Shine-Dalgarno sequences in the mRNA instead of Kozak sequences [8, 9]. These observations suggest that archaeal DNA replication is a process catalyzed by eukaryotic-like proteins in a bacterial context [10]. In addition, there are specific features which belong only to the domain* Archaea*, such as the DNA polymerase D, which add another level of complexity and uniqueness to these microorganisms. While a number of evolutionary hypotheses have been proposed to account for the mosaic nature of this process [11, 12], comparative genomics and ultrastructural studies support two alternative scenarios. The archezoan scenario holds that an archaeal ancestor developed a nucleus and evolved into a primitive archezoan, which later acquired an *α*-proteobacterium to form the mitochondrion and evolved into the eukaryotes. The symbiogenesis scenario holds that an archaeal ancestor plus an *α*-proteobacterium formed a chimeric cell, which then evolved into the eukaryotic cell [12]. A major difference between these hypotheses is the time when the first eukaryotes evolved. In the archezoan scenario, the ancestors of the early eukaryotes are as ancient as the two prokaryotic lineages, the Bacteria and Archaea. In the symbiogenesis scenario, the eukaryotes evolved relatively late after the diversification of the ancient archaeal and bacterial lineages.

The cultivated* Archaea* are taxonomically divided into five phyla.* Crenarchaeota* and* Euryarchaeota* are best characterized, and this taxonomic division is strongly supported by comparative genomics. A number of genes with key roles in chromosome structure and DNA replication are present in one phylum but absent in the other. Crenarchaeotes also exhibit a vast physiological diversity, including aerobes and anaerobes, fermenters, chemoheterotrophs, and chemolithotrophs [2]. The majority of the cultivated crenarchaeotes are also thermophiles [13]. Euryarchaeotes exhibit an even larger diversity, with several different extremophiles among their ranks in addition to large numbers of mesophilic microorganisms. Interestingly, all the known methanogens belong to this phylum. Additionally, environmental sequencing, 16S rRNA analysis, and cultivation efforts provide strong evidence for three additional phyla:* Thaumarchaeota*,* Korarchaeota,* and* Aigarchaeota* [14–16]. A sixth phylum has also been proposed previously, the* Nanoarchaeota*. This phylum includes the obligate symbiont* Nanoarchaeum equitans*, which has a greatly reduced genome [17]. However, a more complete analysis of its genome suggests that* N. equitans* corresponds to a rapidly evolving euryarchaeal lineage and not an early archaeal phylum [18].

## 2. General Overview of the DNA Replication Process

The general process of DNA replication is conserved among the three domains of life, but each domain possesses some functional modifications and variations in key proteins. DNA replication may be divided into four main steps. Step 1 (Preinitiation) starts when specific proteins recognize and bind the origin of replication, forming a protein-DNA complex. This complex recruits an ATP-dependent helicase to unwind the double-stranded DNA (dsDNA). The single-stranded DNA (ssDNA) formed is then protected by ssDNA-binding proteins (SSB). Step 2 (Initiation) corresponds to the recruitment of the DNA primase and DNA polymerase. Step 3 (Elongation) corresponds to the duplication of both strands of DNA at the same time by the same unidirectional replication machine. Because of the antiparallel nature of the DNA, the leading strand is copied continuously, and the lagging strand is copied discontinuously as Okazaki fragments [10]. During replication, a sliding-clamp protein surrounds the DNA and binds to the polymerase to increase processivity or the number of nucleotides added to the growing strand before disassociation of the polymerase from the template. Step 4 (Maturation) corresponds to the completion of the discontinuous replication in the lagging strand by the actions of RNase, DNA ligase, and Flap endonuclease. The variability of the players involved in each of these processes between the three domains of life and more specifically within the* Archaea* domain is discussed in further detail below.

## 3. DNA Replication Components

### 3.1. Preinitiation (Origin of Replication and Origin Recognition)

The number of origins of replication in a genome is correlated with phylogeny and varies among the different domains [19]. Members of the* Bacteria* domain usually possess only one replication origin, but eukaryotic chromosomes contain multiple replication origins. This difference was generally accepted as a clear divisor in DNA replication of eukaryotes and prokaryotes. However, members of the* Archaea* domain display either one or multiple origins of replication. The origins of replication in archaea are commonly AT-rich regions which possess conserved sequences called origin of recognition boxes (ORB) and are well conserved across many archaeal species. Also, smaller versions of the ORBs, called mini-ORBs, have been identified [20]. Members of the* Crenarchaeota* phylum display multiple origins of replication. For example, species belonging to the* Sulfolobales* order contain three origins of replication [20, 21]. Likewise, two and four origins of replication have been found in members of the* Desulfurococcales* (*Aeropyrum pernix*) and* Thermoproteales* (*Pyrobaculum calidifontis*), respectively [22, 23]. In addition to shortening the time required for replication, multiple origins may play a role in counteracting DNA damage during hyperthermophilic growth [24]. In contrast, most members of the* Euryarchaeota* phylum seem to possess only one origin of replication. For example, species belonging to the* Thermococcales* (*Pyrococcus abyssi*) and* Archaeoglobales *(*Archaeoglobus fulgidus*) orders contain only one origin of replication [25–27]. However, members of the order* Halobacteriales* (e.g.,* Halobacterium* sp. NRC-1,* Haloferax volcanii,* and* Haloarcula hispanica*) possess numerous origins of replication that may be part of an intricate replication initiation system [28–30]. In fact, a recent report demonstrated that the different origins of replication in* Haloarcula hispanica* are controlled by diverse mechanisms, which suggest a high level of coordination between the multiple origins [31]. In contrast, the deletion of a single origin of replication in* Haloferax volcanii* affects replication dynamics and growth, but the simultaneous deletion of all four origins of replication accelerated growth compared to the wild type strain [32]. These results suggest that initiation of replication in* Haloferax volcanii* may be dependent of homologous recombination, as it is in some viruses, and generate questions about the purpose of the replication origins [32]. With the exception of* Methanothermobacter thermautotrophicus*, the DNA replication origin has not been experimentally demonstrated in the methanogens [33]. However, bioinformatics analysis by GC-skew in* Methanosarcina acetivorans* [34] and *Z*-curve analysis for* Methanocaldococcus jannaschii* and* Methanosarcina mazei* suggest a single origin of replication. A similar analysis of* M. maripaludis* S2 was inconclusive [35]. The fact that the order* Halobacteriales* is the only euryarchaeote that displays multiple origin of replication suggests that there may have been a lineage-specific duplication in this group. Moreover, the number of origins is not highly conserved even within a single archaeal phylum.

**Figure 1:**
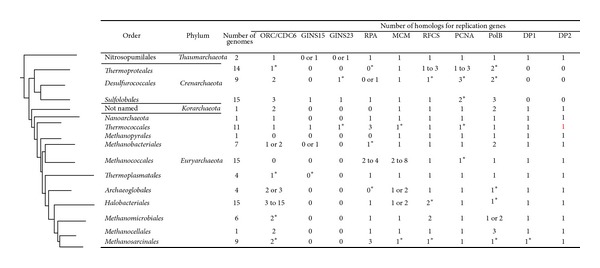
Homologs for some key genes involved in archaeal DNA replication. Archaeal orders are phylogenetically organized following a rooted maximum likelihood tree of* Archaea* based on 53 concatenated ribosomal proteins [36]. The homology search was performed by RAST v4.0 (Rapid Annotation using Subsystem Technology), and the annotated data was viewed through the SEED viewer (http://www.theseed.org/). A total of 115 archaeal genome sequences were obtained from NCBI and uploaded into the RAST server. RAST annotation was performed using default parameters with the genetic code for* Bacteria* and* Archaea*. The replication proteins homologs were checked through the SEED subsystem DNA replication. Homology results of* Thermococcus kodakaraensis* KOD1,* Pyrococcus furiosus* DSM 3638, and* Thermofilum pendens* Hrk 5 were searched for replication protein homologs using BLAST (blastx v2.2.28+). Results with homology coverage of >80% and *E*-values less than 0.001 were considered as real homologs. The results were also supplemented with data from literature reviews and BLAST searches. When indicated in the figure, zero (0) homologs means that no homolog was found for a specific gene by using the described methodology. Asterisks indicate that one or two of the analyzed microorganisms possess an exception for that specific feature. Exceptions noted are (feature/order) ORC1/CDC6/*Thermoproteales, Thermofilum pendens* Hrk5 (3 homologs), and* Thermosphaera aggregans* DSM11486 (2 homologs); ORC1/CDC6/*Thermoplasmatales* and* Picrophilus torridus* DSM 9790 (1 homolog); ORC1/CDC6/*Methanomicrobiales and Methanoplanus petrolearius* DSM11571 (4 homologs); ORC1/CDC6/*Methanosarcinales and Methanosalsum zhilinae* DSM4017 (4 homologs); GINS15/*Thermoplasmatales and Thermoplasma acidophilum* DSM1728 (1 homolog); GINS23/*Desulfurococcales*,* Staphylothermus hellenicus* DSM12710 (no homolog), and* Staphylothermus marinus* F1 (no homolog); GINS23/*Thermococcales*,* Pyrococcus horikoshii* OT3 (2 homologs), RPA/*Thermoproteales, Thermofilum pendens* Hrk5 (3 homologs), and* Thermosphaera aggregans* DSM11486 (1 homolog); RPA/*Methanobacteriales and Methanothermus fervidus* DSM2088 (no homolog); RPA/*Archaeoglobales and Archaeoglobus fulgidus* DSM4304 (1 homolog); MCM/*Thermococcales and Thermococcus kodakaraensis* KOD1 (3 homologs); MCM/*Methanosarcinales and Methanosarcina acetivorans* C2A (2 homologs); RFCS/*Desulfurococcales and Hyperthermus butylicus* DSM 5456 (2 homologs); RFCS/*Halobacteriales and Haloquadratum walsbyi* DSM16790 (1 homolog); RFCS/*Methanosarcinales, Methanosaeta concilii* CG6 (2 homologs), and* Methanosaeta thermophila* PT (2 homologs); PCNA/*Desulfurococcales and Ignisphaera aggregans* DSM17230 (1 homolog); PCNA/*Sulfolobales and Metallosphaera cuprina* Ar-4 (1 homolog); PCNA/*Thermococcales, Pyrococcus horikoshii* OT3 (2 homologs), and* Thermococcus kodakaraensis* KOD1 (2 homologs); PCNA/*Methanococcales, Methanococcus maripaludis* S2 (2 homologs), and* Methanotorris igneus* Kol5 (2 homologs). PolB/*Thermoproteales, Caldivirga maquilingensis* IC-167 (3 homologs), and* Pyrobaculum calidifontis* JCM 11548 (3 homologs); PolB/*Desulfurococcales, Staphylothermus hellenicus* DSM12710 (3 homologs), and* Staphylothermus marinus* F1 (3 homologs); PolB/*Archaeoglobales, Archaeoglobus fulgidus* DSM4304 (2 homologs); PolB/*Halobacteriales, Halorhabdus utahensis* DSM12940 (2 homologs); PolB/*Methanosarcinales, Methanosaeta concilii* GP6 (2 homologs); DP1/*Methanosarcinales, Methanococcoides burtonii* DSM6242 (2 homologs).

The origin of replication is recognized by specific proteins that bind and melt the dsDNA and assist in the loading of the replicative helicases. In* Bacteria* this protein is DnaA, which binds to the DnaA boxes [37]. In eukaryotes, a protein complex known as ORC (origin recognition complex) composed of 6 different proteins (Orc1-6) binds at the replication origin and recruits other proteins [38]. In* Archaea*, the candidate for replication initiation is a protein that shares homology with the eukaryotic Orc1 and Cdc6 (cell division cycle 6) proteins and is encoded by almost all archaeal genomes [39]. Their genes are commonly located adjacent to the origin of replication. These proteins, termed Orc1/Cdc6 homologs, have been studied in detail in representatives of the* Euryarchaeota* (*Pyrococcus*) and* Crenarchaeota* (*Sulfolobus*) and have been shown to bind to the ORB region with high specificity [20, 26, 40]. In addition, purified Orc1/Cdc6 from* S. solfataricus* binds to ORB elements from other crenarchaeotes and euryarchaeotes* in vitro*, suggesting that these proteins recognize sequences conserved across the archaeal domain [20]. The crystal structures of these proteins from* Pyrobaculum aerophilum* [41] and* Aeropyrum pernix* [42] have been solved, contributing to the overall understanding of their activity during the initiation of replication (reviewed in [43]). In general, the number of Orc1/Cdc6 homologs found in archaeal genomes varies between one and three (Figure 1). However, there are some interesting exceptions. Members of the order* Halobacteriales* possess between 3 and 15 Orc1/Cdc6 homologs, which may reflect the large number of origins of replication. Interestingly, like other members of the* Methanosarcinales*, the extremely halophilic methanogen* Methanohalobium evestigatum* Z-7303 only possesses two Orc1/Cdc6 homologs. Thus, the ability to grow at extremely high concentrations of salt and to maintain high intracellular salt concentrations does not necessarily require a large number of Orc1/Cdc6 homologs. Although most members of the order* Methanomicrobiales *possess two copies of this gene,* Methanoplanus petrolearius *DSM 11571 possesses four copies. Thus, the number of Orc1/Cdc6 homologs varies considerably among the euryarchaeotes. In an extreme case, representatives of the orders* Methanococcales* and* Methanopyrales* do not possess any recognizable homologs for the Orc1/Cdc6 protein [7, 44], and the mechanics of replication initiation in these archaea remain an intriguing unknown of the archaeal replication process.

### 3.2. Initiation (DNA Unwinding and Primer Synthesis)

After Orc1/Cdc6 proteins bind to the origin of replication, a helicase is recruited to unwind the dsDNA. In* Bacteria* the replicative helicase is the homohexamer DnaB. In eukaryotes the most likely candidate for the replicative DNA helicase is the MCM (minichromosome maintenance) complex, which is a heterohexamer that is activated when associated with other replicative proteins, forming the CMG (Cdc45-MCM-GINS) complex [45–48]. As in eukaryotes, in* Archaea* the most probable candidate for the replicative helicase is the MCM complex. Intriguingly, the MCM complexes in eukaryotes and archaea possess a 3′-5′ unwinding polarity, but the bacterial DnaB helicase unwinds duplex DNA on the opposite direction with a distinct 5′-3′ polarity [49–51].

Most archaeal genomes studied so far encode one MCM homolog. Its helicase activity has been demonstrated* in vitro* for several archaea, including* Methanothermobacter thermautotrophicus *ΔH, where the MCM protein forms a dimer of hexamers [52, 53]. The* in vivo* interaction between the MCM complex and the Orc1/Cdc6 proteins has been demonstrated in other* Archaea* [54–56]. Indeed, through an* in vitro* recruiting assay, Orc1/CDC6 from* P. furiosus* has been demonstrated as the possible recruiter of the MCM complex to the origin of replication [57]. A recent study identified thirteen species of archaea with multiple* mcm* genes encoding the MCM complexes (Figure 1, [58]). The number of* mcm* homologs is especially high in the order* Methanococcales*, where different representatives possess from 2 to 8 copies [59, 60]. For instance,* Methanococcus maripaludis* S2 possesses four homologs of the MCM protein [44]. Through a shotgun proteomic study, peptides for three of them have been detected [61], suggesting that multiple MCMs are expressed and functional. However, a genome-wide survey of gene functionality in* M. maripaludis* demonstrated that only one of these genes, MMP0030, was likely to be essential for growth [62]. In addition, coexpression of recombinant MCMs from* M. maripaludis* S2 allowed copurification of all four proteins [59], suggesting that* M. maripaludis* may form a heterologous multimeric MCM complex. However, because MMP0030 protein is the only* mcm* gene essential for growth, a homologous multimeric complex may also be possible. Similar results have been found in* Thermococcus kodakaraensis*, in which three MCM homologs are present but only one is essential [63]. The* T. kodakaraensis* MCM system is suggested to form homologous multimeric complexes [64]. A recent study proposed that two of the MCM homologs that are conserved among representatives of the order* Methanococcales* are a consequence of an ancient duplication that occurred prior to the divergence of this group [59]. It has also been proposed that the large number of MCM homologs in the order* Methanococcales* is a direct consequence of mobile elements, which may have taken advantage of the ancient duplication of the MCM genes to take over the replication system by forming cellular MCM heterocomplexes [58]. In any case, the large number of MCM homologs in the order* Methanococcales* may be a product of an intricate and complex evolutionary history. Whether or not it is related to the absence of the replication initiation protein Orc1/Cdc6 is an interesting possibility [58].

In eukaryotes the MCM complex is not active on its own and requires the association of two accessory factors, the heterotetrameric GINS complex (from the Japanese go-ishi-ni-san meaning 5-1-2-3, after the four subunits Sld5, Psf1, Psf2, and Psf3) and the Cdc45 protein. This complex, called CMG (Cdc45-MCM-GINS), is thought to be the active replicative helicase [47]. Homologs for GINS subunits have been identified in* Archaea* by bioinformatics analysis. One homolog is most similar to the eukaryotic proteins Psf2 and Psf3 (GINS23) and is common in the crenarchaeotes. Another homolog is most similar to the eukaryotic proteins Psf1 and Sld5 (GINS15) and is found largely in the euryarchaeotes [65]. The GINS complex is expected to be essential for DNA replication, but the distribution of these two different GINS homologs suggests that the presence of one of them is sufficient for archaeal DNA replication. Some* Crenarchaeota* (*S. solfataricus*) and some* Euryarchaeota* (*P. furiosus *and* T. kodakaraensis*) possess both homologs and form heterotetramers similar to the ones found in eukaryotes with a ratio 2 : 2 [66–68]. To find homologs of GINS15 and GINS23 in* Archaea*, two known GINS15 (from* Sulfolobus solfataricus* P2 and* Thermococcus gammatolerans* EJ3) and three known GINS23 (from* Sulfolobus solfataricus* P2,* Pyrococcus yayanosii* CH1, and* Thermococcus gammatolerans* EJ3) were used for homology searches. These analyses failed to identify GINS homologs in several archaeal groups (Figure 1). These results suggest that there are other unknown GINS homologs in archaea or that the sequences have diverged enough between genera not to be recognized by homology searches. In any case, a more comprehensive analysis of GINS proteins is necessary to fully understand their function and distribution in* Archaea*.

The detailed interactions of the GINS and MCM proteins in archaea appear to be highly variable. Although the* S. solfataricus* GINS and MCM proteins physically interact* in vitro*, MCM helicase activity was not stimulated in the complex [66]. In contrast, the MCM helicase activity of* T. kodakaraensis* and* P. furiosus* is clearly stimulated by their GINS complexes [64, 67]. The crystal structure of the GINS complex of* T. kodakaraensis* was recently determined. The backbone structure and the assembly are similar to the human complex with some notable differences [68]. Interestingly, many other euryarchaeotes possess only the homolog to GINS15. One of those,* Thermoplasma acidophilum,* forms a homotetrameric complex [68, 69]. Moreover,* in vitro *the* T. acidophilum* MCM helicase activity was not affected by the GINS complex [69]. These results suggest that other proteins may be involved in the formation of a stable helicase in many* Archaea*.* M. maripaludis* S2 possesses a hypothetical protein which may correspond to the gene encoding for GINS15, which is probably essential for growth [60, 62]. No homologs of GINS23 are present in the genome of* M. maripaludis.*

The replication related Cdc45 protein is ubiquitous in eukaryotes, but its exact role has not yet been elucidated. Interestingly, homologs of the Cdc45 protein are not present in* Archaea*. However, a bioinformatics analysis revealed that the eukaryotic Cdc45 and the prokaryotic RecJ, which is a conserved 5′-3′exonuclease in most bacteria and archaea, possess a common ancestry and share a homologous DHH domain [70]. These results suggest that the archaeal RecJ may substitute for the eukaryotic Cdc45 during replication. Circumstantial evidence supports this conclusion. In* S. solfataricus*, a homolog of the DNA binding domain of RecJ was purified together with a GINS protein [66]. Similarly, in* T. kodakaraensis*, the RecJ homolog (TK1252p) copurified with proteins of the GINS complex [71] and formed a stable* in vitro* association with the GINS complex [72]. Two RecJ homologs are found in* M. jannaschii* (MJ0977 and 0831), and they partially complement a* recJ* mutation in* E. coli*. The recombinant MJ0977 also possesses high levels of thermostable single-stranded DNA degrading activity similar to RecJ [73].* In M. maripaludis* S2, four proteins possess the DHH domain with similarity to the* M. jannaschii* RecJ homologs (MMP1682, 0547, 1078, and 1314). However, none of them was essential for growth, suggesting that the activities were redundant [62]. Alternatively, another protein, which has a low similarity with* M. jannaschii* RecJ homolog (MMP0285) but possesses the DHH domain, was possibly essential for growth [62]. However, this protein possesses other domains related to transport, which make it an unlikely candidate for RecJ. These results are not definitive, and more experimental data is needed to assign a function to this protein.

As soon as the DNA is unwound, ssDNA is protected from nucleases, chemical modification, and other disruptions by ssDNA binding proteins. These proteins are present in all three domains of life and are called SSB in* Bacteria*, where they form homotetramers or homodimers [74, 75], and RPA (replication protein A) in eukaryotes, where they form a stable heterotrimer composed of 70, 32, and 14 kDa proteins [76]. Although all ssDNA binding proteins contain different combinations of the oligonucleotide/oligosaccharide-binding fold or OB-fold [77], the sequence similarity is low among RPA and the bacterial SSBs [43]. In* Archaea*, different types of ssDNA binding proteins have been reported. In crenarchaeotes, an ssDNA binding protein is only well characterized in* S. solfataricus*. The* S. solfataricus* ssDNA binding protein contains only one OB-fold and can adopt a heterotetramer conformation [78]. However, the protein also appears to exist as a monomer [79]. This protein appears to be more similar to the bacterial proteins in sequence, but its structure is more similar to that of the eukaryotic RPAs [43, 80]. While homologs are recognizable in other members of the* Sulfolobales* and most other* Archaea*, they are absent from the genomes of many members of the closely related order* Thermoproteales* with some few exceptions (Figure 1) [81].

RPAs have been studied from different representatives of the euryarchaeotes, revealing additional diversity.* M. jannaschii* and* M. thermautotrophicus* possess a single RPA subunit, which shares amino acid similarity with the eukaryotic RPA70 and possesses four and five OB-folds, respectively [82, 83].* P. furiosus* possesses three different RPA subunits, which form a stable heterotrimer that is involved in homologous recombination* in vitro* [84]. The* P. furiosus* RPA, therefore, is similar in subunit organization to the eukaryotic RPA. The other members of the* Thermococcales* genera also possess three RPA homologs, which likely form the homotrimeric complex described for* P. furiosus* (Figure 1).* Methanosarcina acetivorans* also possesses three different RPA proteins (RPA1-3), with four, two, and two OB-folds, respectively. However, they do not interact with each other and appear to function as homodimers [85]. This arrangement is common among the* Methanosarcinales* but is not found in the closely related methanogen orders such as the* Methanocellales* (Figure 1). Members of the order* Methanococcales* possess two to four RPA homologs. The genome of* M. maripaludis* S2 contains three possible RPA homologs (MMP0122, 0616, and 1032) [60]. Only MMP0616 and 1032 were likely to be essential for growth [62], suggesting a possible different complex configuration for RPA proteins in this archaeon. It has been hypothesized that the diversity in OB-folds in the archaeal RPAs is a direct consequence of homologous recombination [86].

DNA synthesis starts with the production of a RNA primer, since the DNA polymerases that replicate genomes are incapable of* de novo* DNA synthesis. The RNA primer is synthesized by a DNA-dependent RNA polymerase or primase. DnaG, a single subunit protein, is the primase in* Bacteria*. In eukaryotes, a two-subunit primase, consisting of a small catalytic subunit (PriS) and a large subunit (PriL), is found in a complex with DNA polymerase *α* and its accessory B subunit [87]. In* Archaea*, the first biochemically characterized primase was the eukaryotic primase-like small subunit PriS in* M. jannaschii* [88]. Later, homologs of the PriS and PriL subunits were described in several* Pyrococcus* species [89–93]. The eukaryotic-like DNA primase has also been characterized in the crenarchaeote* S. solfataricus* [94, 95], where it has been shown to interact with MCM through GINS23 [66]. A unique polymerization activity across discontinuous DNA templates has been characterized in the PriSL of* S. solfataricus*, suggesting that this primase may be involved in double-stranded break repair in* Archaea* [96]. So far the eukaryotic-like primases found in archaea exhibit similar properties. PriS functions as the catalytic subunit and PriL modulate its activity [87]. In addition, the archaeal eukaryotic-like primase has the unique ability to synthesize both DNA and RNA* in vitro*. Indeed, the* P. furiosus* PriS synthesizes long DNA fragments, but the addition of PriL regulates the process by decreasing the DNA polymerase activity, increasing the RNA polymerase activity, and decreasing the product length [90]. Additionally, studies of the* P. abyssi* enzyme suggest that DNA primase may also be involved in DNA repair because of the DNA polymerase, gap filling, and strand displacement activities also present* in vitro* [93]. Interestingly, the archaeal primase small subunit resembles DNA polymerases from the Pol X family in sequence and structure, suggesting a similarity in their catalytic mechanism [96].

Homologs of the bacterial DnaG primase are also found in archaea. However,* in vitro* studies of the* P. furiosus* enzyme failed to detect primer synthesis activity, and the* T. kodakaraensis* DnaG was copurified with proteins of the exosome [71]. Also,* in S. solfataricus*, DnaG primase was found to be a core exosome subunit involved in RNA degradation [97, 98]. However, recent studies demonstrated that the* S. solfataricus* DnaG homolog has limited primer synthesis activity, and a dual primase system with PriSL has been proposed to function during DNA replication [99]. Hu et al. hypothesized an interesting theory which suggests that LUCA employed a dual-primase system comprising DnaG and PriSL, which also served roles in RNA degradation and the nonhomologous end joining (NHEJ) pathway. The system was inherited by all three domains. In the* Archaea* domain, it retained its original functions. However, in the* Bacteria* domain DnaG became the major replicative primase and PriSL evolved into the Pol domain of LigD involved in the NHEJ pathway. In contrast, DnaG was lost in the* Eukaryota* domain, and PriSL became the only replicative primase. In addition, it also evolved into the DNA Pol X family involved in NHEJ [96].

Like other* Archaea*,* M. maripaludis* S2 possesses both the eukaryotic- and bacterial-like primases. A new genome-wide survey of this methanoarchaea revealed that genes encoding both subunits of the eukaryotic like primase, PriS, and PriL (MMP0009 and MMP0071) were likely to be essential for growth, which is consistent with a role in replication. Similar observations have been demonstrated in other* Archaea*, such as* Halobacterium *sp. NRC-1and* Haloferax volcanii* [93, 100]. In contrast the DnaG primase (MMP1286) was nonessential, which would be consistent with a role in the exosome [62]. These results suggest that in* M. maripaludis* and probably other euryarchaeotes, a dual primase system is not present.

### 3.3. Elongation (Replicative DNA Polymerase)

The primer synthesized by the primase is further extended by a replicative DNA polymerase. DNA polymerases have been classified into seven families based on their amino acid sequence similarity (A, B, C, D, E, X, and Y) [101–105]. In* Bacteria*,* E. coli* possesses five DNA polymerases, Pol I–V, where the first three belong to families A, B, and C, respectively, and the last two belong to the Y family. The major replicative DNA polymerase in* E. coli* is Pol III, a member of the C family, which synthesizes DNA with high processivity when functioning within the holoenzyme [106]. In eukaryotes, a great diversity of DNA polymerases is also found, but DNA replication requires three replicative DNA polymerases which belong to family B (Pol*α*, Pol*δ*, and Pol*ε*) [107]. In* Archaea*, fewer types of DNA polymerases are present, but they have an interesting evolutionary division. Representatives of the DNA polymerase B family are found in all* Archaea*. The D family DNA polymerase is present in every phyla studied so far with the exception of the* Crenarchaeota* (Figure 1). Lastly, a member of the Y family has been identified and biochemically characterized in* S. solfataricus *[108] and the* Methanosarcinales*, in which several species harbor two homologs [109]. Even though the activity of DNA polymerases belonging to the Y family in archaea is not fully understood, they are suggested to play an important role in DNA repair [109].

Representatives of the DNA polymerase family B have been identified in all* Archaea*. The crenarchaeotes possess at least two family B DNA polymerases (PolB I and PolB II, and in some cases PolB III) [102, 110, 111]. The recombinant* S. solfataricus*,* Pyrodictium occultum,* and* A. pernix* enzymes have been characterized [102]. In contrast, euryarchaeotes only contain PolB I. The recombinant* P. furiosus* enzyme has been characterized [112]. The PolB I enzymes have similar amino acid sequence and overall structure, and they possess a potent 3′-5′exonuclease proofreading activity [102]. A unique property of the archaeal family B DNA polymerase is the ability to stall DNA polymerization in the presence of uracil or hypoxanthine deaminated bases [113, 114]; this property helps to prevent the copy of template-strand uracil and the transmission of fixed mutations to progeny [115]. Commonly, cytosine deamination converts G:C base pairs into the promutagenic G:U mismatches, which result in 50% of the offspring containing an A:T transition mutation after replication [116]. In addition, when A-U base pairs are formed, the loss of the 5-methyl moiety upon replacement of thymine can exert a detrimental effect on protein DNA interactions. Interestingly, in some crenarchaeotes and euryarchaeotes, one of the B family DNA polymerase paralogs possesses disrupted versions of the sequence motifs that are essential for catalysis. Possibly, these enzymes possess a structural role [117].

DNA polymerase family D (PolD) is a novel enzyme that was originally discovered in* P. furiosus* [118]. It has since then been identified in all euryarchaeotes [119]. For a long time, this enzyme was considered a euryarchaeote-specific polymerase, but the three newly discovered archaeal phyla were also found to possess genes for PolD [14–16]. PolD is a heterodimer with a small subunit (DP1) and a large subunit (DP2). It has been proposed that the large subunit harbors the polymerase activity, although its sequence is very different from other hitherto described DNA polymerases. The small subunit possesses high similarity with the noncatalytic B-subunits of the eukaryotic DNA polymerases *α*, *δ*, and *ɛ* [120]. Studies of the* M. jannaschii* enzyme demonstrated that the small subunit possesses a strong 3′-5′exonuclease activity, suggesting that it may be involved in proofreading activity [7, 121]. Efficient polymerase and proofreading activity have also been detected from a purified PolD of* P. furiosus*, and the residues Asp-1122 and Asp-1124 are essential for the polymerization reaction in* P. horikoshii* [122, 123]. Every euryarchaeote examined possessed one homolog for DP1 and DP2, except for* Methanococcoides burtonii* DSM 6242, which possesses two homologs for DP1 (Figure 1).

Because the crenarchaeotes only possess family B DNA polymerases, one of them must be the replicative polymerase. However, in euryarchaeotes it was not clear which enzyme was involved in replication. Studies in* Halobacterium* sp. NRC-1 showed that both PolB and PolD were essential for viability, and it was proposed that they could be working together at the replication fork, synthesizing the leading and lagging strand, respectively [100, 124]. However, in other* Euryarchaeota*, PolB does not appear to be essential. For instance, a recent report demonstrated that both subunits of PolD were essential, but PolB was nonessential for the growth of* M. maripaludis* S2 [62]. Likewise, in* T. kodakaraensis* PolD can be coisolated with different proteins of the archaeal replication fork, but PolB was mainly coisolated with proteins of unknown function [71]. A deletion of the PolB in* T. kodakaraensis* also had no detectable effect on cell viability [125]. Interestingly,* T. kodakaraensis* PolB mutants have increased sensitivity to UV radiation. These results together suggest that PolD is the essential replicative DNA polymerase in these euryarchaeotes and not a PolB family polymerase. In this scenario, PolB may have some other crucial function in* Halobacterium* and possibly other closely related species, which it is not conserved throughout the phylum. Finally, it has been demonstrated that purified PolD from* P. abyssi* is able to perform strand displacement and RNA primer extension* in vitro*. Purified PolB, on its own, cannot perform these activities* in vitro*, but in the presence of PCNA strand displacement activity is stimulated [124, 126]. A recent* in vitro* study in* P. abyssi* demonstrated that RNase HII is required to initiate strand displacement DNA synthesis by PolB [127].

### 3.4. Elongation (Other Accessory Proteins)

Purified DNA polymerases possess low processivity; however, the addition of an accessory factor, the sliding clamp, gives DNA polymerases the required processivity to replicate genomes. This factor, whose structure resembles a doughnut, functions as a molecular platform which recruits several replication-associated enzymes and act together with DNA and the polymerase to stabilize their interactions during replication [128]. The structure of sliding clamps is conserved in the three domains of life. In* Bacteria*, the sliding clamp or *β*-subunit is a homodimeric ring [129]. In eukaryotes, the sliding clamp or proliferating cell nuclear antigen (PCNA) is a homotrimeric ring [128]. In* Archaea*, the majority of crenarchaeotes have multiple PCNA homologs which form heterotrimeric rings [130, 131] (Figure 1). In contrast, most euryarchaeotes possess a single PCNA homolog (Figure 1). Interactions of DNA polymerase with the sliding clamp are mediated through a common motif, called the PCNA Interacting Protein (PIP) box [132]. The three dimensional structures of PCNA-PolB and the PolB-PCNA-DNA complex from* P. furiosus* have been solved, shedding light on the interactions between these molecules [133, 134]. In addition, a study demonstrated that the PolD/PCNA and PolB/PCNA interactions require two and one PIP boxes, respectively [135]. Recently, through a protein-interaction network of* P. abyssi*, a previously unknown protein with nuclease activity (Pab0431) and a PIP canonical motif was discovered to interact with PCNA, suggesting a possible involvement in DNA replication [136].* T. kodakaraensis* is the only well-documented example of an euryarchaeote with two homologs for PCNA, TK0535 (PCNA1), and TK0582 (PCNA2). Recent work demonstrated both proteins form stable homotrimeric rings that interact with* T. kodakaraensis* PolB* in vitro* [137]. A more detailed study of the two homologs of PCNA in* T. kodakaraensis* demonstrated that both homologs stimulate* in vitro* the primer extension activity of PolB, but only PCNA1 and not PCNA2 stimulates the same activity of PolD [138]. Also, the same report showed that the* pcna2* gene can be disrupted without causing growth deficiencies, but it was not possible to isolate mutants of* pcna1*. These results suggest that PCNA1 but not PCNA2 is essential for DNA replication [138]. It has been proposed that one of the PCNA genes was acquired by lateral gene transfer [139]. Members of the order* Methanococcales* possess one PCNA homolog with two exceptions.* M. maripaludis* S2 possesses two PCNA homologs (MMP1126 and 1711) [60]. Both genes appeared to be essential for growth, suggesting that they both play an important role in replication [62]. The gene MMP1711 possesses high similarity to both* T. kodakaraensis* genes and is likely the true methanococcal PCNA. In contrast, MMP1126 possesses only low similarity to the* T. kodakaraensis* genes and also contains an S-adenosylmethionine-dependent methyltransferases domain. Thus, it is likely to possess some alternative function.* Methanotorris igneus* Kol 5 also possesses two PCNA homologs.

For PCNA to assemble around DNA, a specific loading factor is required. In* Bacteria* the loading factor is known as *γ*-complex and comprises three different subunits in a *γ*_3_-*δ*-*δ*′ stoichiometry [140]. In eukaryotes, the loading factor is known as Replication Factor C (RFC) and is a heteropentameric complex comprising one large subunit and four different small subunits [141]. In contrast, in* Archaea* the RFC consists of two different proteins, a small subunit RFCS and a large subunit RFCL. They also form a heteropentameric complex in a 4 : 1 ratio (RFCS : RFCL). Interestingly, many crenarchaeotes possess one homolog of RFCL and two or three homologs of RFCS. Alternatively, most euryarchaeotes possess only one RFCL and RFCS homolog. The exceptions are some members of the orders* Halobacteriales, Methanomicrobiales,* and* Methanosarcinales*, which possess two RFCS homologs (Figure 1). Structural and biochemical studies of RFC have been conducted in several euryarchaeotes, such as* Archaeoglobus fulgidus* and* Pyrococcus* species [142–144]. An interesting case is observed in* Methanosarcina acetivorans*. This RFC possesses three subunits (RFC1, 2, L) found in a ratio 3 : 1 : 1 [145]. Homologs for these three subunits are also present in most of the genomes of the haloarchaea [145]. It is inferred that this type of RFC complex represents an intermediary form transitional between the canonical archaeal RFC complex of one small and one large subunit and the eukaryotic RFC complex of four different small and one large subunit [146]. The organization and spatial distribution exhibit a similarity to the* E. coli* minimal *γ*-complex, but the function of the subunits is probably not conserved [145].

### 3.5. Maturation

During DNA replication, the lagging strand is discontinuously synthesized by extending the RNA primers or Okazaki fragments. This discontinuously synthesized DNA strand requires maturation to form a single, covalently closed strand to end the replication process. In* Archaea*, Okazaki fragments were first demonstrated during replication in* P. abyssi* and* Sulfolobus acidocaldarius* [147]. During replication, these RNA primers are replaced with DNA. The removal of the primers is performed by the enzyme RNase H, which is ubiquitous in the three domains of life. According to their sequence similarity, in prokaryotes the RNase H proteins are classified into three groups: RNase HI, HII, and HIII. In eukaryotes, they are classified as RNase H1 and H2 [148]. However, phylogenetic analyses suggest that the RNase H proteins can also be classified into two different groups: Type 1 (prokaryotic RNase HI, eukaryotic RNase H1, and viral RNase H) and Type 2 (prokaryotic RNase HII and HIII and eukaryotic RNase H2) [149]. These two types of RNase H possess different specific activities, metal ion preferences, and cleavage sites [150]. Originally it was thought that* Archaea* only possess type 2 RNase H [149], but several type 1 archaeal RNase H enzymes have since then been discovered [151, 152]. Interestingly, in eukaryotes, RNase H1 and H2 tend to coexist, and different combinations of the three prokaryotic RNases H (I, II, and III) are found, except for the combination of RNases HI and HIII. This mutually exclusive evolution of some of the prokaryotic RNases seems to be related to functional redundancy [148].

Another enzyme involved in primer replacement in DNA replication is the Flap endonuclease I (FEN-1), which recognizes double-stranded DNA with an unannealed 5′-flap, and cleaves it. A eukaryotic homolog of FEN-1 is found in every archaeal member analyzed, and one member of the* Thermoproteales*,* Thermofilum pendens* Hrk5, possesses two homologs.

*M. maripaludis* S2 possesses genes for the prokaryotic RNase HI and HII, and for FEN-1, but none are essential, suggesting that they may be redundant in their functions [44, 62]. Possibly, both RNase H proteins persist and are evolutionary stable in the genome. The gene products may perform the same function but one is less efficient than the other [153].

After the Okazaki fragments are replaced by DNA in the lagging strand, the nick between the newly synthesized and the elongated DNA is repaired by a DNA ligase. This enzyme uses a nucleotide cofactor to catalyze the formation of the phosphodiester bond in three well-characterized steps [154]. The enzyme is common to all three domains but can be grouped into two families based on cofactor specificity (ATP or NAD^+^). The DNA ligases from several crenarchaeotes and euryarchaeotes have been further characterized (reviewed in [43]). Many archaeal DNA ligases possess dual cofactor specificity (ATP/NAD^+^ or ATP/ADP), but every archaeal DNA ligase characterized so far uses ATP. A thermophilic DNA ligase from* M. thermautotrophicus* which uses ATP as sole cofactor was characterized* in vitro* [155]. A characterized DNA ligase from the crenarchaeote* Sulfophobococcus zilligii* displayed specificity for three cofactors (ATP/NAD^+^/GTP) [156]. Major structural work in this enzyme has been achieved in* P. furiosus* and* S. solfataricus*, and the findings have been reviewed in detail [43]. In* M. maripaludis* S2, the DNA ligase is likely to be essential for growth [44, 62].

Understanding the maturation process in archaea has become a focus of increasing research in recent years. An* in vitro* study reconstituted the Okazaki fragment maturation using proteins derived from the crenarchaeote* S. solfataricus*. They demonstrated that only six proteins are necessary for coupled DNA synthesis, RNA primer removal, and DNA ligation. In this model a single PCNA (heterotrimeric ring) coordinates the activities of PolBI, FEN-1, and DNA ligase into an efficient maturation complex [157]. A different* in vitro* study in* P. abyssi* has demonstrated two different models for Okazaki fragments maturation, which differ in the RNA primer removal process. In the first model RNA primer elimination is executed by continuous PolD strand displacement DNA synthesis and 5′RNA flap cleavage by FEN-1. The resulting nicks are closed by DNA ligase. Alternatively, the second model proposes that RNase HII cleaves the RNA primer, followed by the action of FEN-1 and strand displacement DNA synthesis by polB or polD. The nick is then sealed by DNA ligase [127]. Testing these models* in vivo* is of great interest to complete the picture of how these processes behave in the cell. In addition, other factors that can modulate these processes may be required for coordination of the* in vivo* maturation process.

## 4. Conclusions

DNA replication in archaea possesses a dual nature, where the machinery is structurally and functionally similar to the eukaryotic replication system, but it is executed within a bacterial context [11, 25]. Additionally, unique archaeal features demonstrate the complexity of this process, and it does not appear to be just a simplified version of the eukaryotic system. For example, the archaeal specific DNA polymerase D is conserved across the* Archaea* domain with the exception of the* Crenarchaeota* phylum. The recent discovery that it is the essential replicative polymerase in two different euryarchaeotic species demonstrates an unanticipated variability in archaeal DNA replication and a fundamental difference in the replication mechanism between crenarchaeotes and euryarchaeotes. Interestingly, the absence of PolD in crenarchaeotes is not the only difference in DNA replication. These differences include the absence of histones in crenarchaeotes [4], the presence of multiple origins of replication in crenarchaeotes and a single origin of replication in most euryarchaeotes, the absence of GINS23 in most euryarchaeotes, the absence of RPA protein in many crenarchaeotes, the presence of multiple MCM homologs in* Methanococcales*, and the presence of one homolog of PCNA in euryarchaeotes compared to the multiple homologs in crenarchaeotes. These distinctive characteristics between the phyla highlight the complexity of archaeal DNA replication and suggest a complex evolutionary history (Table 1).

Among the* Euryarchaeota*, the orders* Halobacteriales* and* Methanococcales* possess differences in the DNA replication system that make them unique. For instance,* Halobacteriales* possess many more origin recognition proteins (Orc1/Cdc6) compared to the rest of the archaea. On the other hand,* Methanococcales* and* Methanopyrales* lack recognizable homologs for the Orc1/Cdc6 proteins, suggesting the presence of a very different mechanism for initiation of replication. In addition, the* Methanococcales* possess a large number of MCM protein homologs. It has been proposed that these distinctive features are connected, and because of the absence of the Orc1/Cdc6 proteins, MCM proteins may interact with other unknown initiation enzymes, resulting in a complex phylogeny of the MCM homologs [49]. Presumably, these differences also account for the inability to recognize the origin of replication in these microorganisms. This unique scenario for initiation of DNA replication may be a direct consequence of various processes such as duplication, mobile genetic elements, and interactions with viruses [58].

The differences in DNA replication among archaeal higher taxa demonstrate an unexpected variability and suggest two alternative evolutionary models. In the first model, DNA replication evolved late after the diversification of the archaeal lineages. Because the replicative system was not fully formed, it was possible to develop differently between the lineages. Once formed, replication would then be highly conserved. However, this model is not consistent with differences observed between lineages that must have formed relatively late, such as those between the orders or even, in some cases, within certain orders. The alternative model is that DNA replication has changed throughout the evolution of the archaea. Thus, differences in the replicative systems may represent ancient as well as modern adaptations to changing environments. In this case, differences in the replicative systems may provide important insights into the evolutionary pressures in play during different episodes of archaeal evolution. From this perspective, the differences between the eukaryotic and archaeal replicative systems may not be evidence for an ancient origin of the eukaryotes. Instead, it is entirely plausible that the replicative system in eukaryotes could have evolved relatively late from a well-developed archaeal system.

## Figures and Tables

**Table 1:** DNA replication proteins and features in the domains *Bacteria*, *Eukaryota,* and the two major phyla of the *Archaea* domain. Modified from [43].

DNA replication stage	Process	Bacteria	Eukaryota	Archaea	
				*Crenarchaeota *	*Euryarchaeota *
Preinitiation	Origin of replication	Single	Multiple	Multiple	Single^a^
	Origin recognition	DnaA	ORC complex (ORC 1-6)	Orc1/Cdc6	Orc1/Cdc6^b^

Initiation	DNA unwinding (Helicase)	DnaB	MCM complex (MCM 2-7)	MCM complex	MCM complex
	DNA unwinding (Accessory proteins)	DnaC	Cdc6	GINS23/GINS15	GINS15^c^
			Cdt1		
			GINS complex (Sld5, Psf1-3)	RecJ homolog?	RecJ homolog?
			Cdc45		
	Primer synthesis	DnaG	Pol *α*/primase complex	DNA primase (PriSL)/DnaG^d^	DNA primase (PriSL)

Elongation	DNA synthesis (polymerase)	Pol III (Family C DNA polymerase)	Pol*δ*, and Pol*ε* (Family B DNA polymerase)	Family B DNA polymerase	Family D DNA polymerase^e^
	DNA synthesis (Processivity factors)	*γ*-complex (clamp loader)	RFC (clamp loader)	RFC (clamp loader)	RFC (clamp loader)
		*β*-clamp (clamp)	PCNA (clamp)	PCNA (clamp)	PCNA (clamp)

Maturation	Maturation (Okazaki fragment processing)	Pol I (family A DNA polymerase)	Fen1/Dna2	Fen1	Fen1
		RNase H	RNase H	RNase H	RNase H
		DNA ligase	DNA ligase	DNA ligase	DNA ligase

^a^Exception, the order *Halobacteriales.*

^b^Not known for members of the *Euryarchaeota *orders *Methanococcales* and *Methanopyrales.*

^c^GINS23 has been founded only in the order *Thermococcales* of the *Euryarchaeota*.

^d^ *Sulfolobus solfataricus *did show primase activity *in vitro.*

^e^Family B DNA polymerase is also essential in *Halobacterium*. Because its function has not been clearly elucidated, it might also play a role in replication in this and closely related organisms.
